# Apigenin remodels the gut microbiota to ameliorate ulcerative colitis

**DOI:** 10.3389/fnut.2022.1062961

**Published:** 2022-12-16

**Authors:** Rongrong Fu, Lechen Wang, Ying Meng, Wenqing Xue, Jingjie Liang, Zimu Peng, Jing Meng, Min Zhang

**Affiliations:** ^1^State Key Laboratory of Food Nutrition and Safety, College of Food Science and Engineering, Tianjin University of Science and Technology, Tianjin, China; ^2^Department of Rehabilitation Medicine, Shandong Provincial Third Hospital, Shandong University, Jinan, Shandong, China; ^3^Tianjin International Joint Academy of Biomedicine, Tianjin, China; ^4^China-Russia Agricultural Processing Joint Laboratory, Tianjin Agricultural University, Tianjin, China

**Keywords:** apigenin, ulcerative colitis, anti-inflammatory, gut microbiota, gut barrier integrity

## Abstract

**Introduction:**

Ulcerative colitis (UC), a chronic non-specific colorectal inflammatory disease with unclear etiology, has long plagued human health. Gut microbiota dysbiosis destroy homeostasis of the colon, which is closely related to ulcerative colitis progress. Apigenin, a flavonoid widely present in celery, has been found to improve ulcerative colitis. However, the potential molecular mechanism of apigenin ameliorating ulcerative colitis through protecting intestinal barrier and regulating gut microbiota remains undefined.

**Methods:**

Dextran sodium sulfate (DSS)-induced colitis mouse model was conducted to evaluate the effect of apigenin on UC. Disease activity index score of mice, colon tissue pathological, cytokines analysis, intestinal tight junction proteins expression, and colonic content short-chain fatty acids (SCFAs) and 16S rRNA gene sequencing were conducted to reflect the protection of apigenin on UC.

**Results:**

The results indicated that apigenin significantly relieved the intestinal pathological injury, increased goblet cells quantity and mucin secretion, promoted anti-inflammatory cytokines IL-10 expression, and inhibited the expression of proinflammatory cytokines, TNF-α, IL-1β, IL-6 and MPO activity of colon tissue. Apigenin increased ZO-1, claudin-1 and occludin expressions to restore the integrity of the intestinal barrier. Moreover, apigenin remodeled the disordered gut microbiota by regulating the abundance of *Akkermansia*, *Turicibacter*, *Klebsiella*, *Romboutsia*, etc., and its metabolites (SCFAs), attenuating DSS-induced colon injury. We also investigated the effect of apigenin supplementation on potential metabolic pathways of gut microbiota.

**Conclusion:**

Apigenin effectively ameliorated DSS-induced UC *via* balancing gut microbiome to inhibit inflammation and protect gut barrier. With low toxicity and high efficiency, apigenin might serve as a potential therapeutic strategy for the treatment of UC via regulating the interaction and mechanism between host and microorganism.

## 1 Introduction

Inflammatory bowel diseases (IBDs) are complex, chronic gastrointestinal inflammatory diseases such as Crohn’s disease (CD) and ulcerative colitis (UC) (1). Hereditary, immune response, and environmental factors are the potential pathogenesis for IBD (2). UC is a chronic non-specific colorectal inflammatory disease, which has a high global incidence (3). The clinical symptoms of UC are abdominal pain, diarrhea, and hematuria. Furthermore, the permeability of the intestinal barrier and the levels of inflammatory cytokines are significantly increased (4). Therefore, it was made one of the most challenging gastrointestinal diseases.

As a sophisticated microbial ecosystem, the gut microbiome has a great influence on the health of the host intestine through dietary fiber fermentation and necessary metabolites. In patients with UC, biodiversity in the gut microbial composition decreased, which manifested as a reduction of beneficial bacteria, for instance, *Faecalibacterium* and other butyrate producers (5). Short-chain fatty acids (SCFAs), the small molecule metabolic end-product of gut microbiota, are produced in the intestine through the fermentation of proteins and complex carbohydrates. It is confirmed that SCFAs can inhibit intestinal inflammation (6). Mucins, largely secreted by goblet cells, contain many immunomodulatory molecules, thereby protecting the host from invading pathogens (7). *Lactobacillus* spp. and *Akkermansia muciniphila* are important bacteria maintaining intestinal mucin homeostasis (8). Therefore, modulating gut microbiota to relieve intestinal inflammation is of clinical importance in UC studies.

Apigenin, a natural plant bioactive compound, is widely found in many fruits, vegetables, and herbs, such as celery. Increasing evidence has proved that apigenin has many biological and pharmacological activities, which can modulate cell proliferation and apoptosis (9), inflammation (10), and cancer development (11). Previous studies revealed that apigenin affects the growth of gut microbiota in mice, and it might be degraded by gut microbiota (12–14). However, the exact underlying mechanism of apigenin in the treatment of UC by modulating gut microbiota has not been clarified.

In this study, to evaluate the effect of apigenin administration on UC, we employed a 2% dextran sulfate sodium (DSS)-induced UC mouse model combined with apigenin intervention. Furthermore, we explored whether apigenin supplementation could alleviate colitis by inhibiting the inflammatory response, protecting the gut barrier, and balancing the gut microbiome structure in colitis mice.

## 2 Materials and methods

### 2.1 Animals and induction UC

C57BL/6 mice (6–8 weeks of age, weighing 16–18 g) were acquired from the SPF Biotechnology Co., Ltd. (Beijing, China). Before the experiment, the mice were given 3 days to adapt to the laboratory environment. Throughout the adaptation and study periods, all animals were maintained on a 12-h light-dark cycle (23 ± 1°C with a relatively constant humidity of 50 ± 5%) under specific pathogen-free (SPF) conditions. The animal study was carried out according to the guidelines of the Animal Experimentation Ethics Committee, Tianjin University of Science and Technology.

All the mice were randomly divided into five groups, namely, the control group, model group, positive medicine group (SASP), low-dose apigenin (AP-L) group, and high-dose apigenin (AP-H) group (*n* = 6 per group). The mice in the control group drank water normally, and experimental mice were administered 2.0% (w/v) dextran sodium sulfate (DSS, MW, 36–50 kDa; Meilunbio, Dalian, China) in drinking water for 23 days to induce experimental colitis. After 2 days, mice in the model groups received 0.5% of CMC-Na, mice in the positive medicine group received 200 mg/kg of SASP, and mice in AP-L and AP-H groups received 150 and 250 mg/kg of apigenin, respectively, which was given by intragastric administration once a day for 21 days.

### 2.2 Sample collection

During the experiment, mice in each group were weighed every 2 days, and feces were collected to observe their traits. Fecal consistency, fecal occult blood, and weight loss were used for Disease Activity Index (DAI) scores. Mice were sacrificed under 0.5% pentobarbital sodium (50 mg/kg, I.P.) on day 26. Blood was centrifuged at 4,000 r/min at 4°C for 20 min, and serum was stored at −80°C for further analysis. The general morphology of colon samples was photographed. The length of the colon was measured. Colonic contents were collected and stored at −80°C. Colon tissues were fixed in 10% formalin for further analysis.

### 2.3 H&E and AB-PAS staining

The colon samples were fixed, dehydrated, and embedded in paraffin. Tissues embedded in paraffin were cut into 3 μm sections. Before staining, the sections were deparaffinized and rehydrated. Hematoxylin-eosin (H&E) staining used standard techniques. Eosin (ZSGB-BIO, Beijing, China) was used to stain cytoplasm, and hematoxylin (ZSGB-BIO) was used to stain the nucleus. Alcian blue periodic acid-Schiff (AB-PAS) (LEAGENE, Beijing, China) staining was performed to measure the goblet cell quantity of colon tissue. The sections were stained with alcian blue for 15 min and oxidized with periodic acid for 5 min. Then, the sections were stained with Schiff dye for 15 min. All tissue sections were observed using an optical microscope (Olympus, Tokyo, Japan). Mucosal damage and inflammatory cell infiltration were used as study parameters to evaluate the histological scores.

### 2.4 ELISA

Colon tissues were ground with physiological saline and centrifuged (3,000 rpm, 20 min) at 4°C. The supernatant was collected for further analysis. Cytokines including TNF-α, IL-6, IL-1β, and IL-10 in colonic tissues were measured by ELISA Kits (mlbio, Shanghai, China) according to the manufacturer’s instructions. Absorbance values were detected with a microplate reader. The cytokine concentration was obtained according to the standard curve.

### 2.5 Myeloperoxidase

The level of myeloperoxidase (MPO) in colonic tissues was measured by an MPO activity assay kit (mlbio, Shanghai, China). The detection method was carried out according to the manufacturer’s instructions, and the absorbance value was measured at 460 nm.

### 2.6 Western blotting

Colon tissues were washed with phosphate-buffered saline (PBS) and lysed fully in ice-cold lysis buffer with the Protease Inhibitor Cocktail (MCE, New Jersey, USA) on ice. Lysates were separated by electrophoresis and transferred onto polyvinylidene difluoride membranes (Millipore, Massachusetts, USA). The membranes were blocked with 5% skim milk powder and incubated with primary antibody against ZO-1 (1:1,000, Bioss), Occludin (1:500, Bioss, Beijing, China), Claudin1 (1:1,000, Bioss), and GAPDH (1:25,000, Proteintech, Chicago, USA) at 4°C overnight and then incubated with a horseradish peroxidase-conjugated goat anti-mouse/rabbit IgG secondary antibody (1:5,000, YEASEN, Shanghai, China) for 1 h at room temperature. The intensity of protein was assessed using a chemiluminescent imaging system (Image Quant LAS 4000, Atlanta, USA).

### 2.7 Immunohistochemistry staining and analysis

Immunohistochemistry (IHC) was conducted according to the previously former method (15). Colon tissue embedded in paraffin was sliced (3 μm) and then deparaffinized and rehydrated with dimethyl-benzene and alcohol. Next, samples were incubated with 3% H_2_O_2_ for 10 min to block endogenous peroxidase activity and then blocked with goat serum (Proteintech, Chicago, USA) for 1 h. After being blocked, the samples were incubated with the following primary antibodies overnight at 4°C: ZO-1 (1:250, Bioss, Beijing, China), Occludin (1:400, Bioss), and Claudin1 (1:200, Bioss). The tissues were washed three times with PBS for 5 min each time. After that, the primary antibody in the negative group was displaced by PBS. The secondary antibody was subsequently added with the horseradish peroxidase-polymer anti-mouse/rabbit IHC kit (Maixin Biotech, Fuzhou, China) for 1 h at room temperature. 3,3′-Diaminobenzidine-tetrahydrochloride (DAB) was used to stain the tissues and counterstained with hematoxylin. All the samples were observed with a microscope (Olympus, Tokyo, Japan). The immunohistochemistry score was calculated by multiplying the intensity (0 = negative, 1 = canary yellow, 2 = claybank, 3 = brown) and the positive cell percentage scores (1 = less than 25%, 2 = 25–50%, 3 = 51–75%, 4 = more than 75%).

### 2.8 Short-chain fatty acids

The short-chain fatty acids in the colonic contents were measured by GC-MS (SHIMADZU, GCMS-QP2010 Ultral, Kyoto, Japan). The method of SCFA determination is according to a previously described method (16). After the fecal samples were dissolved and homogenized, the mixture of sulfuric acid and ether was added to extract SCFAs and then centrifuged at 4°C, 12,000 rpm for 15 min to prepare the samples. Then, anhydrous sodium sulfate was added to the supernatant and centrifuged at 4°C (12,000 rpm, 15 min). The supernatant was collected and injected into GC-MS. The standard SCFAs are a mixture of acetic acid, propionic acid, butyric acid, isobutyric acid, valeric acid, and isovaleric acid (MACKLIN, Shanghai, China).

### 2.9 16S rRNA gene sequencing

Genomic DNA was extracted from colonic contents. DNA pyrosequencing of the 16S rRNA V3-V4 region was completed in Novogene Co., Ltd. (Beijing, China). The primers 341F and 806R (forward 5′-CCTAYGGGRBGCASCAG-3′ and reward 5′-GGACTACNNGGGTATCTAAT-3′) were used to amplify DNA. Bioinformatics analysis was performed on the Illumina MiSeq platform of Novogene.

### 2.10 Statistical analysis

All data are presented as means ± SD. ANOVA test (one-way or two-way, with *post-hoc* tests) was performed using GraphPad Prism 7.0 software (^*^P ≤ 0.05, ^**^P ≤ 0.01).

## 3 Results

### 3.1 Apigenin protects against UC in mice

**FIGURE 1:**
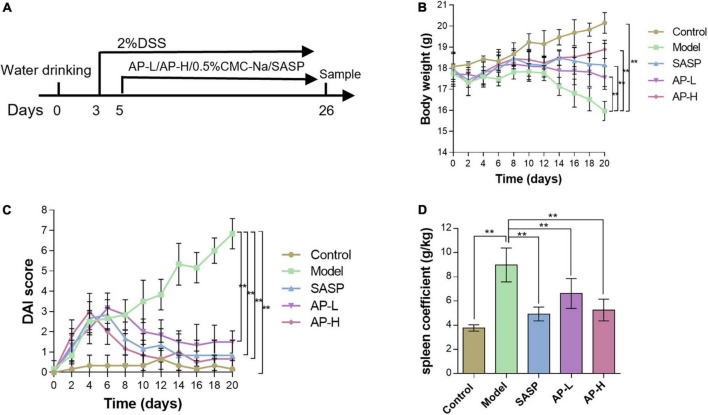
Apigenin protected experimental UC in mice. **(A)** A schematic of the UC and apigenin (AP) treatment design. **(B)** Changes in body weights (g) of mice. **(C)** Disease activity index (DAI) scores for weight loss and fecal status in each group. **(D)** Spleen coefficient of each treatment group. **P < 0.01.

To investigate whether apigenin has a therapeutic effect on UC, mice were treated with 2.0% DSS in drinking water, while apigenin was administered by gavage from day 3 to day 24 (Figure 1A). As expected, DSS markedly reduced the body weight compared to normal mice. In contrast, SASP significantly increased body weights, and apigenin ameliorated weight loss in a dose-dependent manner in mice induced by DSS (Figure 1B) (*P* < 0.01). DAI score based on stool consistency, bloody stool, and weight loss assessments were also evaluated. DSS could significantly increase the DAI, whereas SASP and apigenin treatment could improve this phenomenon (Figure 1C) (*P* < 0.01). In addition, it was reported that DSS-induced colitis developed symptoms of spleen hypertrophy (17). The results showed that apigenin inhibited spleen hypertrophy in colitis mice compared to the mice in the DSS-induced colitis group (Figure 1D) (*P* < 0.01). Overall, these results indicated that apigenin administration treatment significantly ameliorated DSS-induced UC.

### 3.2 Apigenin attenuates DSS-induced colon injury

**FIGURE 2:**
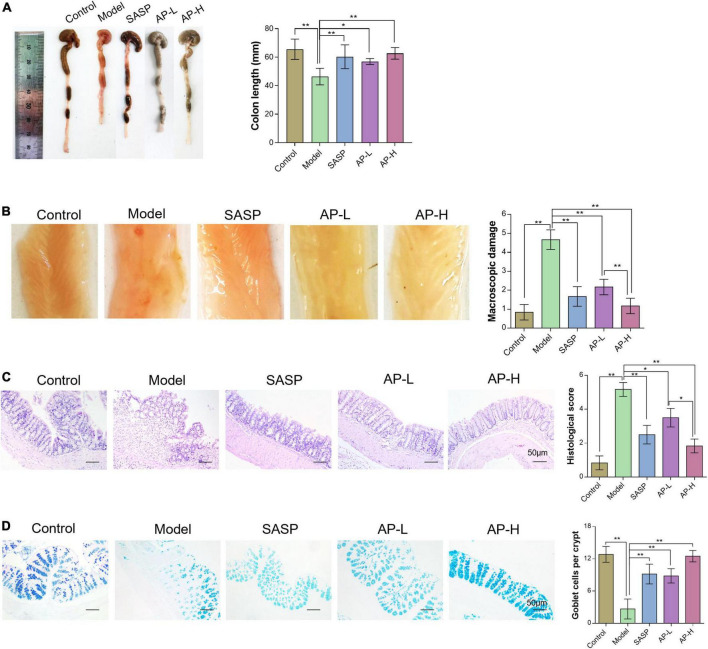
Apigenin attenuated DSS-induced colon injury. **(A)** Representative pictures of colon gross appearance and colon length. **(B)** Colonic morphology and associated macroscopic damage scores in mice. **(C)** H&E staining of the colon in mice and related microscopic lesion scores. **(D)** AB-PAS staining of colon tissues in mice among different groups. *P < 0.05, **P < 0.01.

To further investigate the therapeutic effect of apigenin on UC intuitively, we compared the length, gross morphology, and tissue damage of colorectal sites in each group of mice. DSS typically causes colonic shortening while such change was also improved by 150 and 250 mg/kg of apigenin. Moreover, there was no significant difference in colorectal length between high-dose apigenin-treated mice and SASP-treated mice (Figure 2A) (*P* < 0.05). The colorectal tissue displayed obvious fold, good elasticity, and reflectance in the wild mice. Colon tissues from UC mice displayed indistinguishable upper colorectal folds, granular mucous membranes, multiple hyperemia and swelling, and surface ulcers. Apigenin ameliorated these colonic damages and reduced colon macroscopic damage scores (Figure 2B) (*P* < 0.01). Histological examination of the hematoxylin and eosin-stained sections of the colon revealed severe inflammatory lesions, crypt erosion, and inflammatory cell infiltration from UC mice. Strikingly, SASP and apigenin-treated mice exhibited less inflammatory cell infiltration and intact colonic architecture without mucosal damage, reducing microscopic damage scores (Figure 2C) (*P* < 0.05). AB-PAS staining results demonstrated that compared with the control group, there was a lower goblet cell quantity of colon in model mice, while apigenin administration restored the damage induced by DSS (Figure 2D) (*P* < 0.01). Overall, apigenin supplementation could effectively alleviate the severity of colon injury in mice with UC.

### 3.3 Effect of apigenin on inflammatory cytokines in UC mice

**FIGURE 3:**
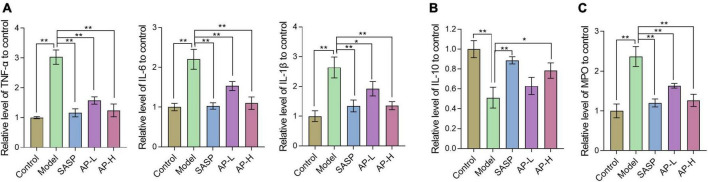
Apigenin treatment altered the cytokine expression in colitis mice. **(A)** Expression levels of inflammatory cytokines TNF-α, IL-6, and IL-1β in colon tissues. **(B)** Expression levels of inflammatory cytokines IL-10 in colon tissues. **(C)** Expression levels of MPO in colon tissues. *P < 0.05, **P < 0.01.

To demonstrate the effect of apigenin on inflammation, inflammatory cytokines (TNF-α, IL-1β, IL-6, and IL-10) were determined. Increased proinflammatory cytokines TNF-α, IL-1β, and IL-6 expressions were observed in the model group. Compared with mice induced by DSS, SASP and apigenin treatment decreased TNF-α, IL-6, and IL-1β levels (Figure 3A) (*P* < 0.05). IL-10 produced by intestinal CD_4_^+^ T cells is responsible for maintaining immune homeostasis, thereby inhibiting colitis development (18). The results showed that apigenin increased the level of IL-10 in the colitis of DSS-induced colitis mice (Figure 3B) (*P* < 0.05). MPO, an enzyme present in neutrophils, is positively correlated with the concentration of neutrophils in the inflammatory region, and increased MPO activity is an indicator of neutrophil infiltration and inflammation (19). In the model group, MPO activity increased, and apigenin treatment inhibited neutrophil infiltration and inflammation in UC mice (Figure 3C) (*P* < 0.01). It is noteworthy that apigenin had an effect on TNF-α, IL-6, IL-1β, IL-10, and MPO in a dose-dependent manner. These results suggested that apigenin alleviated inflammation and kept intestinal homeostasis.

### 3.4 Apigenin increases gut barrier integrity of colitis mice

**FIGURE 4:**
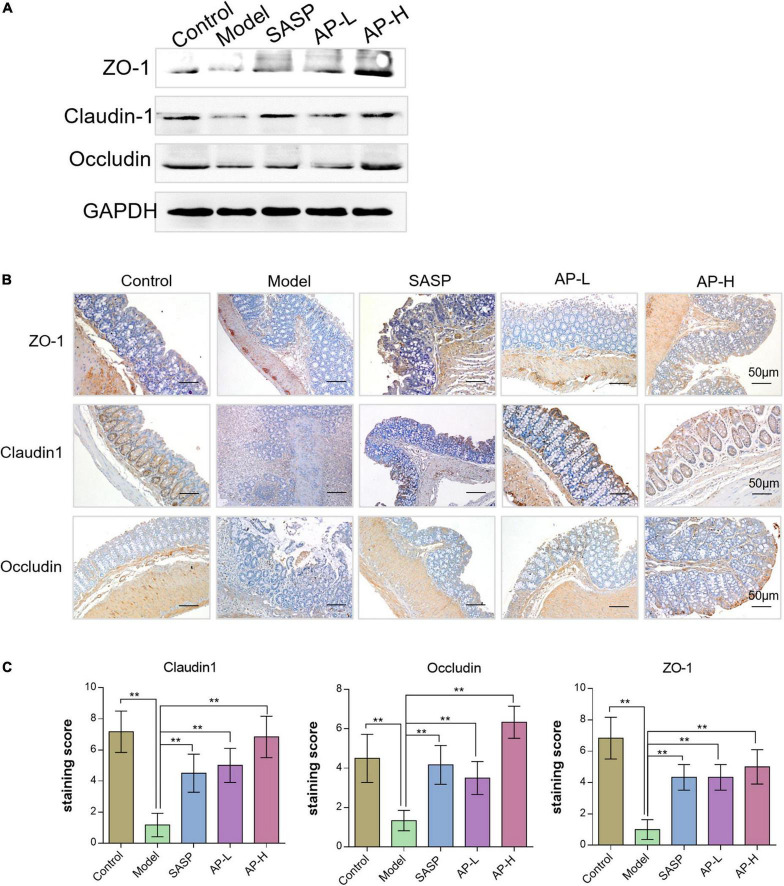
Effects of apigenin on gut barrier integrity in DSS-induced colitis mice. **(A)** Western blot analysis detecting ZO-1, claudin-1, and occludin expressions of colon tissues in each group. **(B)** ZO-1, claudin-1, and occludin expression levels in the colon tissues analyzed through IHC staining. **(C)** IHC staining index of ZO-1, claudin-1, and occludin in colon tissues. **P < 0.01.

Gut barrier damage was the moment pathological characteristic of colitis (2), such as UC. The zonula occluden (ZO), occludin, and claudin family proteins are essential tight junction proteins of epithelial barrier (20). The levels of tight junction proteins were determined through Western blot analysis and IHC analysis. As shown in Figure 4A, the protein expressions of ZO-1, claudin-1, and occludin in the model group were reduced, while the supplement of SASP and apigenin could improve the expression of these proteins. Furthermore, the IHC images and staining scores indicated that substantial loss of staining intensity of ZO-1, claudin-1, and occludin in mice induced by DSS was observed compared with the control group (Figures 4B, C) (*P* < 0.01). However, SASP and apigenin might improve this change, and high-dose apigenin might have a better effect compared with SASP. The above results demonstrated that apigenin prevented gut barrier damage in DSS-induced colitis mice.

### 3.5 Effect of apigenin on gut microbiota and its metabolites in UC mice

**FIGURE 5:**
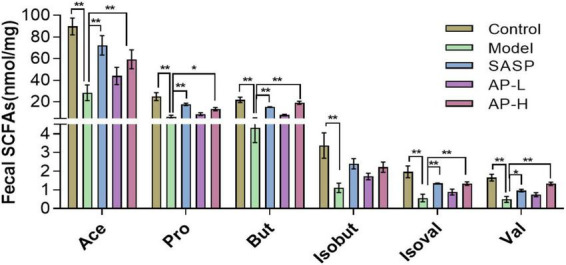
Apigenin treatment improved short-chain fatty acid (SCFA) contents. *P < 0.05, **P < 0.01.

As metabolites of intestinal microflora, SCFAs maintain intestinal function by inhibiting inflammation and protecting the intestinal epithelial barrier (21). We detected the contents of SCFAs using GC-MS. Compared with the control group, the concentration of SCFAs in model groups was downregulated (*P* < 0.01). But apigenin and SASP administration significantly increased the level of SCFAs, and the SCFA content of AP groups was almost as effective as positive drugs (Figure 5) (*P* < 0.05).

**FIGURE 6:**
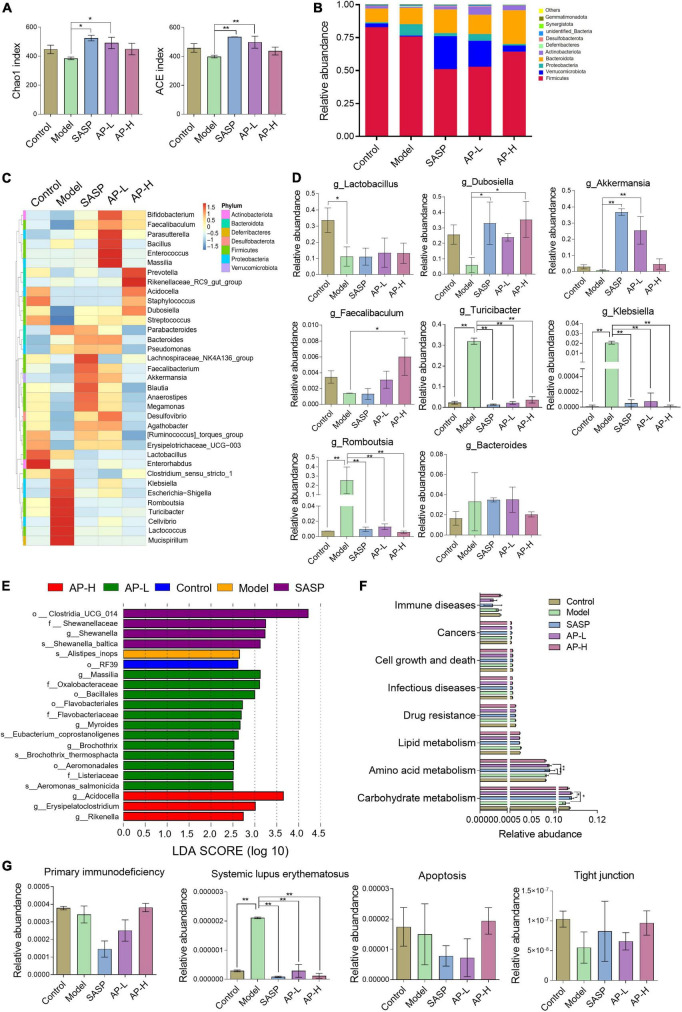
Effect of apigenin on gut microbiota in UC mice. **(A)** Chao1 and ACE index of samples. **(B)** Relative abundance of the top 10 microbial groups at the phylum level. **(C)** Community heatmap at the genus level. **(D)** Relative abundances of dominant bacteria at the genus level between different groups. **(E)** LDA discriminant histogram. **(F)** Microbial catabolism function of KEGG pathways among control, model, SASP, AP-L, and AP-H groups. **(G)** The effect of apigenin administration on primary immunodeficiency, systemic lupus erythematosus, apoptosis, and tight junction. *P < 0.05, **P < 0.01.

To investigate whether apigenin has a moderating effect on the composition and diversity of gut microbiota, we performed 16S rRNA gene sequencing analysis of the colonic content of different groups of mice. Alpha diversity, such as Chao1 and ACE index, was recovered in apigenin and SASP groups (Figure 6A). To explore the specific variations in different groups, we evaluated the relative abundance of taxonomic groups. At the phylum level, we found that the dominant phylum was *Firmicutes*, *Verrucomicrobia*, and *Proteobacteria*. The results showed that DSS induction contributed to decreases in *Firmicutes* and *Verrucomicrobia* and increases in *Proteobacteria*. Apigenin and SASP intervention could significantly reverse the DSS-induced variation in the relative abundance of *Verrucomicrobia* and *Proteobacteria* (Figure 6B).

In the top 35 genus level, 16 genera were downregulated and 12 genera were upregulated in the DSS-induced mice, while SASP and apigenin supplementation selectivity reversed this change (Figure 6C). Among them, the relative abundances of *Lactobacillus*, *Dubosiella*, *Akkermansia*, and *Faecalibaculum* were decreased and *Turicibacter*, *Klebsiella*, *Romboutsia*, and *Bacteroides* were increased at genus level in DSS-induced mice. Apigenin and SASPs administration selectively reversed the relative abundance (Figure 6D). Previous studies proved that *Lactobacillus* (22, 23), *Dubosiella* (24), *Akkermansia* (25, 26), and *Faecalibaculum* (27) are probiotics protecting the intestinal immune barrier from damage. *Turicibacter* (28, 29), *Klebsiella* (30), *Romboutsia* (31), and *Bacteroides*, pernicious bacteria in IBD, might have a positive correlation with the malignant development of UC. In addition, linear discriminant analysis (LDA) showed the main feature of the dominant flora among five different groups (Figure 6E). Our results clarified that DSS treatment altered species diversity of intestinal microbial homeostasis, while apigenin restored that to the normal level.

**FIGURE 7:**
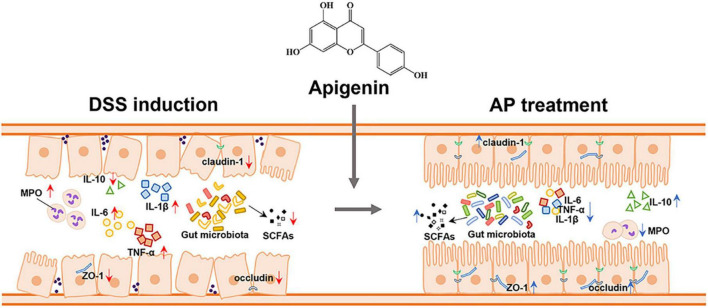
Apigenin effectively ameliorated DSS-induced ulcerative colitis by balancing the gut microbiome structure and its metabolites (SCFAs) to inhibit the inflammatory response and protect the gut barrier.

To investigate the effect of apigenin supplementation on the potential metabolic pathways of gut microbiota in DSS-induced mice, Tax4Fun analysis was executed. As shown in Figure 6F, apigenin notably reversed the changes in many basic metabolic pathways such as carbohydrate metabolism and amino acid metabolism. Furthermore, we analyzed the metabolic changes of gut microbiota related to immune diseases, cell growth, and cell death. The results indicated that apigenin possibly had a close correlation in ameliorating the primary immunodeficiency, systemic lupus erythematosus, apoptosis, and tight junction motivated by DSS (Figure 6G). These findings demonstrated that treatment of apigenin may be effective in restoring dysregulated gut microbiota, which might play its role in the metabolite of bacteria. The protective mechanism of apigenin against UC is shown in Figure 7.

## 4 Discussion

Ulcerative colitis is an inflammatory condition that affects the alimentary canal, and the lesion location mainly includes the colon, rectum, or entire colorectal region (32). Owning to the high gradual incidence and therapeutic drugs with limited side effects on the treatment of patients with UC, the optimal treatment approaches for UC are needed to be explored. In this study, we constructed a UC model in mice treated with 2% DSS for 21 days. The apigenin treatment might suppress inflammatory responses and improve gut barrier damage by regulating the gut microbiome structure.

DSS-induced mice have poor mental states and suffer from weight loss, diarrhea, hematochezia, shorten colon, and splenomegaly (33). In this study, we found that apigenin significantly inhibited the decrease of body weight and colon shortening in mice. Apigenin significantly ameliorated hyperemia, swelling, surface ulcers, and splenomegaly. Moreover, goblet cells located in the intestinal epithelium generate mucin, protecting the intestinal barrier (34). Our results suggested that apigenin and SASP increased the goblet cell quantity and mucin content of colon tissues.

Overexpression of proinflammatory cytokines contributes to intestinal and mucosal inflammation, which can promote neutrophil migration and increase the phagocytic capacity of macrophages (35, 36). Our *in vivo* studies showed that apigenin inhibited TNF-α, IL-6, and IL-1β secretions in colitis of DSS-induced mice. IL-10 inhibits the secretion of proinflammatory cytokines and blocks the differentiation and proliferation of macrophages, thereby preventing excessive immune reaction and tissue damage (19). We found that apigenin increased the IL-10 level, improving the intestine’s immune barrier function. In addition, the results showed that apigenin decreased the MPO level, which inhibited neutrophil infiltration and inflammation in UC mice. Therefore, apigenin restored intestinal barrier function by regulating inflammatory factors and inhibiting neutrophil infiltration in UC.

In the development of colitis, the expression of tight junction proteins, such as ZO-1, occludin, and claudin-1, is negatively correlated with increased intestinal barrier permeability (37). The damage of this barrier is related to the pathogenesis of UC and its clinical symptoms, such as diarrhea and hematochezia. Our data showed that apigenin might have a better therapeutic effect on mechanical barrier integrity by maintaining the expression of ZO-1, claudin-1, and occludin to relieve the UC.

More evidence indicates that the gut microbiome plays a vital role in the development of UC (5). Recent studies showed that severe dysbacteriosis is observed in UC, which exhibits an abnormal abundance of *Firmicutes*, *Bacteroidota*, and *Proteobacteria* (29). Through 16S rRNA gene sequencing, we found that the reduction of *Proteobacteria* was observed by the apigenin and SASP intervention, which indicated that apigenin supplementation may regulate intestinal microbial composition and richness to rebalance gut microbiome. It has been proved that *Lactobacillus* could be profitable in UC treatment (38, 39). Our study showed that DSS induced *Lactobacillus* reduction in UC mice. However, apigenin treatment did not significantly affect the abundance of *Lactobacillus*. Therefore, the combination therapy of supplementing *Lactobacillus* with apigenin may be a potential treatment strategy for UC. Moreover, apigenin supplementation significantly enhanced the abundance of *Akkermansia* and *Faecalibaculum*. Intestinal flora produces kinds of metabolites that interact with intestinal epithelial cells, which are closely related to human disease (40). SCFAs, mainly including acetic acid, propionic acid, and butyric acid, were produced by some beneficial bacteria. Microbiota-derived SCFAs and SCFA supplementation upregulated IL-22 production, which protects intestines from inflammation to maintain intestinal homeostasis (41). Besides, amino acid metabolisms such as tryptophan deficiency and its decreased metabolic derivatives could contribute to the development of IBD (42). UC may be associated with the development of colorectal cancer (43). Function prediction data suggested that DSS-induced UC may result in disequilibrium of immune homeostasis and metabolic changes, such as primary immunodeficiency and apoptosis. Overall, apigenin administration remodeled the intestinal bacterial community to relieve UC. The precise mechanism of apigenin affecting the interaction between gut microbiota and the metabolic products in the UC should be further determined.

In conclusion, our studies indicated that apigenin, a natural functional food component, could effectively ameliorate DSS-induced UC by improving the intestine’s immune barrier function, mechanical barrier integrity, and biological barrier function in UC mice. With the advantages of low toxicity and side effects, apigenin is expected to be a potential therapeutic strategy for patients with UC.

## Data availability statement

The original contributions presented in this study are included in the article/supplementary material, further inquiries can be directed to the corresponding authors.

## Ethics statement

This animal study was reviewed and approved by Animal Experimentation Ethics Committee, Tianjin University of Science and Technology.

## Author contributions

RF, LW, and YM contributed to experimental operation and data analysis. WX and JL contributed to data analysis and wrote sections of the manuscript. ZP conducted sections of experimental operation. JM and MZ arranged subject ideas and provided theoretical, technical guidance, and financial support. All authors contributed to the article and approved the submitted version.
